# Impact of physical, psychological, and social frailty on quality of life by sex in adults aged 65 and older: a secondary analysis of the Korean Longitudinal Study of Aging (2018~2022)

**DOI:** 10.7586/jkbns.25.037

**Published:** 2025-08-29

**Authors:** Hyun-Jeong Kim, Yun-Hee Kim

**Affiliations:** Department of Nursing, Pukyong National University, Busan, Korea

**Keywords:** Frailty, Quality of life, Aged, Sex factors

## Abstract

**Purpose:**

This research aimed to explore the impact of multidimensional frailty—comprising physical, psychological, and social domains—on quality of life (QoL) in adults aged 65 years and older, with an emphasis on sex differences.

**Methods:**

A secondary analysis was conducted using data from the seventh to ninth waves (2018~2022) of the Korean Longitudinal Study of Aging. Frailty was operationalized using proxy indicators conceptually aligned with the physical, psychological, and social domains defined by the Tilburg Frailty Indicator. QoL was evaluated using a self-reported, single-item scale. Generalized estimating equations were used to investigate the relationships between each frailty domain and QoL by sex while controlling for pertinent covariates.

**Results:**

Overall frailty significantly reduced QoL in both men (B = –7.98, *p* < .001) and women (B = –9.42, *p* < .001). Psychological frailty had the greatest negative impact on QoL in men (B = –8.51, *p* < .001), while physical frailty was the most detrimental in women (B = –6.88, *p* < .001). Social frailty had the least impact but remained statistically significant in both sexes.

**Conclusion:**

These results underscore the importance of implementing a multidimensional, sex-sensitive framework for assessing and managing frailty. Nurses should conduct comprehensive frailty evaluations and implement tailored strategies to address each domain of frailty, particularly in community settings. The results also support integrating multidimensional frailty concepts into nursing education and practice to enhance QoL in aging populations.

## INTRODUCTION

Frailty is characterized by increased dependency due to declining physical function, diminished physiological capacity, and reduced adaptability to stressors [1]. Individuals who are classified as frail often experience negative outcomes, such as increased rates of hospitalization, loss of independence, and premature death [2]. Frailty has recently been conceptualized as a multidimensional and fluid phenomenon, incorporating physical, psychological, and social domains, which has led to a broader comprehension of aging-related vulnerability [3]. This expanded perspective aligns with holistic nursing principles and facilitates more comprehensive assessment and care strategies [4]. Managing multidimensional frailty necessitates a holistic perspective that incorporates physical, mental, and social aspects in clinical assessments and care planning [2]. Multidimensional frailty is increasingly understood to reflect impairments across physical, psychological, and social domains, rather than being limited to physical decline alone [5].

Multidimensional frailty consists of three main domains: physical, psychological, and social frailty. First, physical frailty is significantly correlated with lower overall and health-related quality of life (QoL) and is associated with negative consequences, including loss of independence, elevated risk of falls and mortality, increased use of healthcare services, and reduced social engagement [6-10]. Psychological frailty reflects diminished psychological resilience, encompassing depressive symptoms and cognitive impairment [11,12]. Greater severity of depressive symptoms is associated with reduced physical activity and diminished QoL, particularly in social aspects [13,14], while cognitive decline negatively affects instrumental activities of daily living, thereby impairing QoL [15]. Social frailty, which arises from weakened social connections and reduced engagement with the community, includes social isolation, low social support, and poor socioeconomic status [16]. Social frailty has a strong negative correlation with QoL [17] and is also associated with nutritional risk, depression, and cognitive decline [18]. An examination of prior research indicates that comprehensive consideration of frailty is essential when assessing QoL.

According to Statistics Korea, adults aged 65 and older are projected to account for 19.2% of the population in 2024, exceed 20% by 2025, and surpass 30% by 2036 and 40% by 2050 [19]. This transition to a super-aged society highlights the urgency of early identification and management of frailty, especially to prevent QoL deterioration. As of 2023, single-person elderly households numbered approximately 2.14 million, accounting for 37.8% of all older adult households—an increase from 32.9% in 2015 [19]. In the same year, 10.8% of Korean adults aged 19 or older perceived older people as the most vulnerable group to discrimination or human rights violations [19]. This trend reflects not only structural changes in households, but also shifting societal perceptions of aging and vulnerability. One study found that older adults living alone exhibited significantly higher levels of depressive symptoms than those living with others, and that physical inactivity and limited social engagement were significantly associated with depressive symptoms [20]. These demographic and psychosocial factors underscore the necessity of a multidimensional approach to understanding older adults’ QoL

Recent international studies have actively explored the collective influence of physical, psychological, and social frailty on QoL and have reported significant associations across these domains [5,21-23]. In Korea, most frailty research has relied on physical criteria (e.g., Fried phenotype), with limited attention to psychological and social dimensions [18,23-25]. In particular, studies examining the impact of multidimensional frailty on QoL from a sex-specific perspective are notably lacking in Korea, underscoring the need for integrated analyses that reflect sex differences.

Frailty is generally more prevalent among women, potentially due to biological and social vulnerabilities, with studies reporting rates of 15% in women and 11% in men [26,27]. Evidence from international studies reinforces this pattern; for instance, findings from European countries indicate that older women have significantly higher odds of experiencing frailty than older men, with some studies estimating up to a 1.28-fold increased risk among the female population [28,29]. According to previous studies, men tend to report higher QoL scores than women; however, when controlling for covariates, the sex difference in QoL has been reported as statistically nonsignificant [24]. In contrast, other studies have found no significant differences in QoL scores between men and women regardless of frailty levels, indicating inconsistent findings across the literature [23]. Therefore, it is necessary to examine the impact of frailty on QoL in older adults by sex through a more detailed analysis.

Therefore, this study aimed to classify frailty types and analyze their associations with QoL by sex, using nationally representative data from the Korean Longitudinal Study of Aging (KLoSA). While previous studies directly applied the original Tilburg Frailty Indicator (TFI) tool [2], given the constraints of secondary data, proxy variables conceptually aligned with the TFI framework were used to operationalize physical, psychological, and social frailty. The findings aim to support individualized, sex-based nursing strategies and reinforce the importance of addressing multidimensional frailty to improve QoL in older adults. These findings also emphasize the need for the prevention and management of physical, psychological, and social frailty to improve QoL. In this study, we seek to provide foundational evidence to reinforce the relevance of a multidimensional frailty approach and support the development of individualized, sex-based nursing practices for older adults.

## METHODS

### 1. Study design

A secondary analysis of data from the 7th to 9th waves (2018 to 2022) of the KLoSA was conducted to examine how physical, psychological, and social frailty affect QoL in adults aged 65 years and older.

### 2. Participants

**Figure 1.**
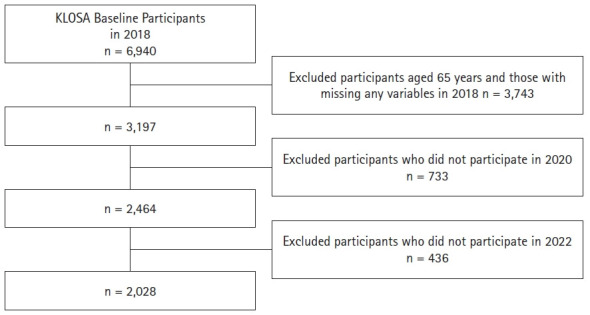
Flowchart of participant selection for the longitudinal analysis (2018~2022). KLoSA = Korea Longitudinal Study of Aging.

This study did not involve primary data collection; instead, it utilized secondary data from the 7th (2018), 8th (2020), and 9th (2022) waves of the KLoSA. Among the 6,940 respondents in the 7th wave of KLoSA, 3,197 individuals were initially selected after excluding those younger than 65 years and those with missing data on any variables included in the analysis (n = 396). Of these, respondents who did not participate in the 8th wave (2020, n = 733) or the 9th wave (2022, n = 436) were further excluded. The final analytic sample consisted of 2,028 individuals who completed all three waves (2018, 2020, and 2022) and provided complete data for all study variables. A total of 6,084 person-wave observations were included in the longitudinal analysis (Figure 1).

### 3. Instruments

#### 1) Frailty

Frailty was assessed using a proxy scale derived from conceptually aligned KLoSA items in accordance with the TFI framework [2]. While the original TFI includes 15 self-assessment items across three domains, this study selected 15 corresponding variables from the KLoSA that were conceptually aligned with the TFI framework. Each item was dichotomized (0 = absence, 1 = presence), and domain-specific and total scores were calculated by summing the respective item values. Overall frailty was defined as a total score of ≥ 5 across all 15 items spanning the three domains [2]. Physical frailty was defined as a score of ≥ 3, psychological frailty as a score of ≥ 2, and social frailty as a score of ≥ 2. These cut-off values were adopted from a previous study [21].

##### (1) Physical frailty

The physical frailty domain included the following eight components: subjective health status, unintentional weight loss, reduced mobility, impaired balance, activity limitations due to hearing or vision problems, low handgrip strength, and fatigue. Each variable was coded using KLoSA data. Subjective health was scored as 1 for “poor” and 0 for all other responses. Weight loss over the past year was scored as 1; weight gain or no change was scored as 0. Fatigue was scored as 1 if experienced for ≥ 3 days in the past week and 0 otherwise. Regular exercise was coded as 0 for “yes” and 1 for “no.” Fall experience and hearing- or vision-related limitations in daily activities were scored as 1 for “yes” and 0 for “no.” Handgrip strength was coded as 1 if it was ≤ 26 kg for men or ≤ 18 kg for women and 0 if it was above the threshold. Since the KLoSA dataset lacks direct measures of mobility and balance, regular exercise and fall experience were used as proxy variables for these components, respectively. This decision was grounded in existing literature indicating that physical inactivity is strongly associated with reduced mobility, and that fall experience is widely recognized as a clinical indicator of impaired postural control and balance [30,31].

##### (2) Psychological frailty

The psychological frailty domain consists of the following four components: cognitive impairment, depressed mood, anxiety, and reduced coping ability. Cognitive impairment was coded as 1 for respondents with a clinical diagnosis of dementia or mild cognitive impairment and 0 otherwise. Depressed mood, trouble sleeping, and feelings of being unable to get going were considered present if experienced for three or more days during the past week and were scored as 1; otherwise, they were scored as 0. Trouble sleeping and the inability to get going were conceptually adopted as proxy variables for anxiety and reduced coping ability, respectively. This substitution was theoretically justified by empirical findings that sleep disturbance is strongly associated with symptoms of anxiety in older adults, and the inability to initiate or sustain activity reflects poor psychological resilience and reduced coping ability [32,33].

##### (3) Social frailty

The social frailty domain included the following three components: living alone, loneliness, and lack of social support. Living alone was coded as 1 for respondents who reported not cohabiting with anyone and 0 otherwise. Loneliness was operationalized using the frequency of contact with close friends: those who interacted less than once or twice per week were scored as 1, and all others as 0. Lack of social support was coded as 1 if the respondent indicated no available person to provide long-term care in the future and 0 if such support was reported. As the KLoSA dataset does not contain a direct item measuring loneliness, the frequency of social interaction was adopted as a conceptually consistent proxy variable for this construct. This approach was supported by prior studies that identify limited frequency of interpersonal interaction as a strong surrogate indicator of perceived social isolation and loneliness [34].

#### 2) QoL

QoL was assessed through a single self-reported item: “Compared to others your age, how satisfied are you with your overall QoL?” Responses were rated on a 0 to 100 scale, with higher scores reflecting greater perceived life satisfaction [24]. QoL was assessed based on a visual analog scale similar to the EuroQol Visual Analogue Scale (EQ-VAS). The EQ-VAS has demonstrated acceptable psychometric properties—including construct validity and applicability across diverse populations—supporting its use as a global health status measure [35].

#### 3) Covariates

Covariates included key sociodemographic and health-related factors. Age was categorized as 65~74 or ≥ 75 years and education as elementary school, middle school, or high school and above. Spouse status was defined as living with a spouse or not. Residential area was classified into metropolitan, urban, or rural regions. Religion, smoking, and alcohol consumption were coded as binary variables (yes/no). Annual personal income was grouped into high (≥ 10.4 million KRW), middle (4.6~< 10.4 million KRW), and low (< 4.6 million KRW). The number of chronic diseases was derived from the presence of the following physician-diagnosed conditions: hypertension, diabetes, malignancy, chronic pulmonary disease, hepatic disease, cardiovascular disease, stroke, arthritis or rheumatism, and gastrointestinal disorders. Participants were then categorized into three groups: none, one, or two or more chronic conditions. To assess multicollinearity, tolerance and variance inflation factor (VIF) values were examined. The tolerance values ranged from 0.79 to 0.98, and all VIF values were between 1.02 and 1.27, remaining well below the conventional threshold of 10. These results indicate that multicollinearity was not a concern in the present study.

### 4. Data collection

The KLoSA targeted middle-aged and older adults aged 45 years and older residing in South Korea, excluding Jeju Island. A total of 10,254 individuals were sampled to establish a nationally representative panel, with an intended sample size of approximately 10,000. The KLoSA is a biennial survey that examines society, family, health status, employment, income and consumption, assets, subjective expectations, and QoL of the population for research purposes. Since 2006, the baseline survey has been conducted biennially using face-to-face computer-assisted personal interviewing with standardized questionnaires [36].

### 5. Data analysis

Statistical analyses were conducted using SPSS version 29.0 (IBM Corp., Armonk, NY, USA). Descriptive analyses were performed to characterize the study population. To identify sex-specific differences in QoL according to sociodemographic factors, independent t-tests and one-way analysis of variance were used, followed by Scheffé’s post-hoc tests, as appropriate. Sensitivity analyses were performed using alternative cut-off points for each frailty domain to assess the robustness of the findings. Generalized estimating equations were applied to assess the impact of physical, psychological, and social frailty on QoL across sexes. This approach was used to appropriately model the correlated nature of repeated QoL measurements, allowing for valid estimation despite within-subject dependency. Subgroup analyses were conducted separately for men and women to examine sex-specific associations between frailty domains and QoL.

### 6. Ethical considerations

This study was approved by the Institutional Review Board of Pukyong National University (IRB No. 2025-02-003). Data from the 7th to 9th waves of the KLoSA, provided by the Korea Employment Information Service, were used. All data were fully anonymized in compliance with the Personal Data Protection Act and Statistical Act to ensure participant confidentiality by preventing the identification of individual respondents.

## RESULTS

### 1. General characteristics and QoL by sex and frailty status

**Figure 2.**
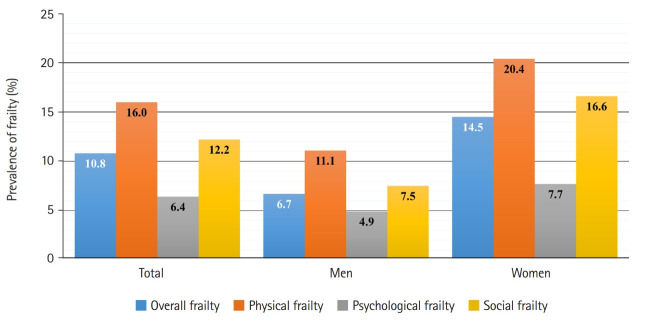
Prevalence of overall, physical, psychological, and social frailty.

The prevalence of frailty among the study participants by sex are presented in Figure 2. The prevalence of overall frailty was 10.8% (men = 6.7%, women = 14.5%). Specifically, the prevalence of physical frailty was 16.0% overall (men = 11.1%, women = 20.4%), psychological frailty was 6.4% (men = 4.9%, women = 7.7%), and social frailty was 12.2% (men = 7.5%, women = 16.6%). Among both men and women, the prevalence was the highest for physical frailty, followed by social frailty and psychological frailty.

Table 1 summarizes the participants’ general characteristics, frailty status, and QoL stratified by sex. Among men, those identified as having overall frailty reported a significantly lower mean QoL score (51.54 ± 21.95) than their non-frail counterparts (66.10 ± 14.01) (t = –5.27, *p* < .001). Those classified as physically frail exhibited significantly lower mean QoL scores of 58.88 ± 19.92 than their non-frailty counterparts (65.76 ± 14.45) (t = –3.53, *p* < .001). Similarly, men with psychological frailty reported a mean QoL score (52.55 ± 21.31), which was significantly lower than that of non-frail men (65.76 ± 14.45) (t = –4.20, *p* < .001). Social frailty in men was associated with a significantly lower QoL score of 55.56 ± 18.91 than in non-frail men (65.89 ± 14.50) (t = –4.53, *p* < .001).

A similar pattern was observed among women. Those with overall frailty exhibited a significantly lower mean QoL score (50.19 ± 17.09) than non-frail women (64.06 ± 14.82) (t = –10.49, *p* < .001). Those with physical frailty reported significantly lower QoL scores (53.27 ± 16.55) than those without frailty (64.03 ± 14.97) (t = –8.93, *p* < .001). Women with psychological frailty exhibited a mean QoL score of 47.44 ± 17.27, which was significantly lower than those without psychological frailty (63.27 ± 15.20) (t = –8.05, *p* < .001). Additionally, social frailty was associated with significantly reduced QoL (57.56 ± 17.44) compared to non-frail women (62.94 ± 15.47) (t = –3.81, *p* < .001). Across all domains, women with any frailty type consistently reported significantly lower QoL than those without frailty.

### 2. Sensitivity analysis of the association between frailty and QoL across different cut-off thresholds

Based on the sensitivity analysis results presented in Table 2, overall frailty showed a significant negative association with QoL across all thresholds. When the cut-off was set at ≥ 4, the association was B = –3.71 (*p* < .001); at ≥ 5, the association was B = –4.90 (*p* < .001); and at ≥ 6, the association was B = –7.25 (*p* < .001), indicating that the impact on QoL increased with higher thresholds. Physical frailty was significantly associated with reduced QoL at all thresholds: B = –2.32 (*p* < .001) at ≥ 2, B = –3.50 (*p* < .001) at ≥ 3, and B = –3.97 (*p* < .001) at ≥ 4. Psychological frailty consistently demonstrated significant associations across all thresholds. At ≥ 1, the association was B = –4.49 (*p* < .001); at ≥ 2, the association was B = –4.70 (*p* < .001); and at ≥ 3, the association was B = –4.34 (*p* = .021). Social frailty was also significantly and negatively associated with QoL at all thresholds: B = –1.78 (*p* < .001) at ≥ 1, B = –1.74 (*p* = .013) at ≥ 2, and B = –2.64 (*p* = .005) at ≥ 3. These findings provide strong evidence for the validity and sensitivity of the main study results, demonstrating that the negative impact of frailty on QoL is consistently observed across a range of cut-off points.

### 3. Association between physical, psychological, and social frailty and QoL by sex

Table 3 presents the associations between frailty subtypes and QoL stratified by sex. Among men, overall frailty was associated with a significant reduction in QoL (B = –7.98, *p* < .001). Among women, the negative impact was even greater (B = –9.42, *p* < .001). In men, psychological frailty had the most substantial negative association with QoL (B = –8.51, *p* < .001), whereas physical frailty (B = –3.02, *p* = .003) and social frailty (B = –2.53, *p* = .035) showed comparatively smaller effects. In contrast, among women, physical frailty demonstrated the strongest association with reduced QoL (B = –6.88, *p* < .001), followed by psychological (B = –5.88, *p* < .001) and social frailty (B = –2.61, *p* = .001). A visual summary of the estimated effects of frailty domains on QoL by sex is presented in Appendix 1.

## DISCUSSION

This study examined the impact of physical, psychological, and social frailty on the QoL among adults aged 65 years and older, using data from the 7th to 9th waves (2018~2022) of the KLoSA, with particular emphasis on sex.

In this study, physical frailty (16.0%) showed the highest prevalence among the three domains; however, the rates of social frailty (12.2%) and psychological frailty (6.4%) were also substantial and should not be overlooked. This result aligns with prior evidence, emphasizing that frailty encompasses a multidimensional phenomenon beyond physical function [37]. In particular, the notably high rate of social frailty suggests that factors such as social isolation, limited support networks, and reduced community engagement may significantly contribute to increased vulnerability in older adults. Accordingly, comprehensive assessment of frailty should incorporate psychological and social components to more accurately identify at-risk individuals and establish holistic intervention strategies aimed at improving health and QoL in aging populations.

Specifically, a sex-stratified analysis further revealed that physical, psychological, and social frailty were observed in 11.1%, 4.9%, and 7.5% of men, and in 20.4%, 7.7%, and 16.6% of women, respectively. According to the study findings, frailty was more prevalent among women than among men across the physical, psychological, and social dimensions. Similar sex disparities have been identified in prior research, which may be attributable to the interplay of biological aging, social disadvantages, and environmental exposure [38]. These results reaffirm that frailty is not merely a physical condition but a complex and multidimensional phenomenon. The notable frequency of social frailty in women underscores the importance of addressing sex-specific vulnerabilities and structural social inequities. Therefore, a comprehensive assessment of frailty must adopt a sex-sensitive approach that integrates psychological and social dimensions to more effectively identify vulnerable individuals and inform holistic intervention strategies.

In this study, overall frailty demonstrated a significant association with lower QoL among both sexes. This aligns with previous findings that overall frailty has a greater impact on QoL than individual domains [2,5]. These findings suggest that frailty in older adults is not limited to physical decline but represents a multidimensional condition affecting overall QoL Furthermore, these results highlight the need for early and comprehensive assessment strategies that integrate physical, psychological, and social frailty. In clinical settings, intensive and individualized interventions are required, particularly for high-risk older adults with concurrent frailty across multiple domains [39]. The prevention of frailty onset and progression is an essential public health goal, especially in super-aged societies. Timely interventions may substantially reduce medical service use and enhance the QoL in older populations. Prior studies have shown that interventions addressing multiple domains, including nutrition, physical activity, and cognitive function, are more effective in improving frailty than those targeting a single domain, with effects sustained for up to 12 months [40]. These findings support the use of multidisciplinary approaches as more sustainable and practical strategies for frailty prevention. Therefore, domain-specific assessments of physical, psychological, and social frailty are essential to guide individualized nursing strategies based on the unique characteristics of each domain.

Our results showed that, among men, psychological frailty had the greatest negative impact on QoL, followed by physical and social frailty. In contrast, among women, physical frailty was the most detrimental, followed psychological and social frailty.

Among older women, physical frailty emerged as the most influential factor impacting QoL decline. This finding may reflect female-specific patterns in physical activity participation and physiological susceptibility, such as a higher prevalence of osteoporosis and sarcopenia and reduced engagement in resistance-based exercise [41]. Physical frailty undermines autonomy by limiting mobility and independence, thereby compromising a core determinant of QoL in older adults [9]. These biological and behavioral factors may contribute to older women’s heightened susceptibility to the detrimental effects of physical frailty. Although it is well-established that physical function in aging can be improved through exercise, the role of resistance training in preserving muscle mass is particularly emphasized [42]. Accordingly, nurse-led, sex-sensitive interventions promoting consistent engagement in resistance and home-based training may be crucial for mitigating physical frailty among older women residing in the community.

Psychological frailty had a significant impact on QoL decline among men. Prior studies have identified depression and cognitive deficits as key factors contributing to diminished life satisfaction in older adults [43,44]. Moreover, sex-based differences in psychological coping strategies have been found to influence QoL outcomes [39]. The pronounced impact of psychological frailty on older men’s QoL may be attributed to emotional processing patterns and coping behaviors commonly observed in this population. Although older men frequently employ task-oriented coping, heightened stress levels may provoke maladaptive responses such as emotional avoidance, alcohol intake, or substance dependence [45,46]. These tendencies may intensify the impact of psychological frailty on their QoL. These findings highlight the need for sex-sensitive psychological interventions. Nurses should conduct regular psychosocial assessments targeting older men who display emotional withdrawal or social isolation. Evidence supports that nurse-led home visits and telephone-based counseling effectively improve mental health, promote social connectedness, and decrease levels of depression in community-dwelling older adults [47].

Although social frailty had the smallest impact on reduced QoL among the frailty domains, it remained significant in both sexes. A similar pattern was observed in a Korean study, where social vulnerability in older adults correlated with reduced satisfaction in life [25] Given its high prevalence and the growing trend of social isolation among older adults, this domain warrants greater attention in both clinical evaluation and public health planning, despite its modest effect size. Findings from systematic reviews indicate that dimensions of social frailty, such as social isolation and loneliness, can adversely impact both mental and physical well-being [48]. This suggests that the relatively weaker impact of social frailty is attributable to its complex interactions with physical and psychological frailty. Additionally, cultural and national differences in social systems, such as family structures, levels of social engagement, and perceptions of social need, may influence how social frailty manifests and affects QoL [49]. To enhance understanding of how social frailty influences QoL, future studies should incorporate the sociocultural context of older adults through repeated, context-specific investigations to validate findings and inform culturally appropriate interventions. Moreover, given their accessibility and trust within communities, nurses are well-positioned to identify socially isolated individuals and connect them with appropriate community-based support services. Integrating social frailty into nursing curricula and training programs would strengthen nurses’ competencies in both recognizing and addressing the unmet social needs of older adults [50]. This integrative approach is expected to not only strengthen the capacity of the nursing workforce but also contribute to holistic, person-centered care addressing multidimensional vulnerabilities of aging populations

This study has some limitations. First, frailty was not directly measured using the original TFI. Instead, proxy variables conceptually aligned with the TFI framework were used to capture physical, psychological, and social frailty, due to the absence of validated frailty tools in the KLoSA dataset. Although this approach enabled multidimensional assessment, it might limit the psychometric validity and generalizability of the findings. Future studies should conduct primary data collection using validated instruments such as the TFI, to ensure conceptual alignment and measurement consistency across frailty domains. Second, QoL was assessed using a single self-reported item rather than a multidimensional, validated instrument, thus warranting cautious interpretation. Researchers are encouraged to adopt multidimensional and psychometrically robust tools—such as the World Health Organization Quality of Life-BREF or the 36-Item Short Form Health Survey—to enable a more comprehensive evaluation of QoL. Finally, while the study identified sex-based differences in the impact of frailty on QoL, the potential interaction or cumulative effects among these domains were not analyzed. The inherently multidimensional and synergistic nature of frailty may have limited the study’s ability to fully capture the complexity of its impact on QoL. The use of advanced statistical approaches—such as interaction modeling and moderation or mediation analysis—may facilitate a more nuanced understanding of how different frailty domains interact and have a combined effect on health outcomes.

Despite these limitations, this study offers several noteworthy strengths. First, by mapping KLoSA items to the physical, psychological, and social domains of the TFI, we demonstrated the feasibility of constructing a theoretically grounded multidimensional frailty index using secondary data. Although formal validation was not performed, this framework-based method offers a practical alternative in the absence of direct frailty measures. Second, as the KLoSA provides nationally representative longitudinal data, these proxy‐defined frailty constructs can be generalized to the Korean older adult population, thereby enabling robust population‐level epidemiologic surveillance. Third, sex‐stratified analyses revealed distinct patterns in how each frailty domain affects QoL, offering critical evidence for sex‐sensitive nursing strategies. Finally, although social frailty exerted the smallest statistical effect on QoL, its comparatively high prevalence underscores the clinical importance of addressing social vulnerability through targeted interventions.

## CONCLUSION

This study demonstrated that multidimensional frailty, including physical, psychological, and social domains, significantly diminishes QoL among Korean older adults. Notably, psychological frailty had the most detrimental effect in men, whereas physical frailty exerted the greatest impact in women, indicating sex-specific patterns in frailty-related QoL decline.

These findings highlight the importance of adopting a multidimensional approach to frailty assessment in gerontological nursing practice. Integrating physical, psychological, and social dimensions allows for a more accurate identification of vulnerable individuals and supports the development of individualized, sex-sensitive nursing interventions. Additionally, this study emphasizes the need for enhanced frailty education within nursing curricula and for the expansion of care strategies to address the complex needs of older adults.

Based on the findings of this study, the following directions are proposed for future research. First, replication studies are needed to evaluate the validity and reliability of the TFI when applied to older adult populations, particularly within Korean contexts. Second, future research should focus on the development and validation of integrated intervention programs that address not only physical but also psychological and social components of frailty, considering sex-specific differences. Third, to enhance generalizability, further studies should include diverse older adult populations beyond community-dwelling individuals, including those in institutional or rural settings. Lastly, advanced analytical approaches such as structural equation modeling or mediation analysis should be employed to investigate the interaction and cumulative effects among the different frailty domains.

## Figures and Tables

**Table 1.** General Characteristics and Quality of Life by Sex and Frailty Status (N = 2,028)

Variables	Categories	Total	Men (n = 965)				Women (n = 1,063)			
				Quality of life	t or F	*p*		Quality of life	t or F	*p*
Overall frailty	Yes	219 (10.8)	65 (6.7)	51.54 ± 21.95	−5.27	< .001	154 (14.5)	50.19 ± 17.09	−10.49	< .001
	No	1,809 (89.2)	900 (93.3)	66.10 ± 14.01			909 (85.5)	64.06 ± 14.82		
Physical frailty	Yes	324 (16.0)	107 (11.1)	58.88 ± 19.92	−3.53	< .001	217 (20.4)	53.27 ± 16.55	−8.93	< .001
	No	1,704 (84.0)	858 (88.9)	65.76 ± 14.45			846 (79.6)	64.03 ± 14.97		
Psychological frailty	Yes	129 (6.4)	47 (4.9)	52.55 ± 21.31	−4.20	< .001	82 (7.7)	47.44 ± 17.27	−8.05	< .001
	No	1,899 (93.6)	918 (95.1)	65.76 ± 14.45			981 (92.3)	63.27 ± 15.20		
Social frailty	Yes	248 (12.2)	72 (7.5)	55.56 ± 18.91	−4.53	< .001	176 (16.6)	57.56 ± 17.44	−3.81	< .001
	No	1,780 (87.8)	893 (92.5)	65.89 ± 14.50			887 (83.4)	62.94 ± 15.47		
Age (year)	65~74	1,212 (59.8)	590 (61.1)	66.61 ± 14.46	3.87	< .001	622 (58.5)	63.30 ± 15.34	3.04	.002
	≥ 75	816 (40.2)	375 (38.9)	62.77 ± 15.81			441 (41.5)	60.29 ± 16.59		
Education level	Elementary school	936 (46.2)	273 (28.3)	61.94 ± 15.96a	11.32	< .001	663 (62.4)	60.17 ± 16.41a	15.44	< .001
	Middle school	415 (20.5)	195 (20.2)	64.26 ± 14.28			220 (20.7)	63.50 ± 14.74		
	High school or above	677 (33.3)	497 (51.5)	67.20 ± 14.63b		(a < b^†^)	180 (16.9)	67.22 ± 14.18b		(a < b^†^)
Spousal status	Yes	1,538 (75.8)	896 (92.8)	65.67 ± 14.61	3.24	< .001	642 (60.4)	63.08 ± 15.77	2.62	.009
	No	490 (24.2)	69 (7.2)	57.97 ± 19.30			421 (39.6)	60.48 ± 16.06		
Residential areas	Metropolitan city	783 (38.6)	371 (38.4)	64.58 ± 15.44	0.90	.405	412 (38.8)	61.63 ± 16.41	1.32	.268
	Small and medium city	659 (32.5)	315 (32.6)	64.86 ± 15.36			344 (32.4)	61.45 ± 16.12		
	Town and village	586 (28.9)	279 (29.0)	66.13 ± 14.37			307 (28.8)	63.29 ± 15.04		
Religion	Yes	829 (40.9)	339 (35.1)	65.69 ± 14.22	−0.89	.373	490 (46.1)	62.92 ± 15.54	−1.64	.101
	No	1,199 (59.1)	626 (64.9)	64.81 ± 15.58			573 (53.9)	61.31 ± 16.23		
Personal income (KRW)	Low (< 4.6 million)	561 (27.7)	132 (13.7)	62.20 ± 14.95a	18.15	< .001	429 (40.4)	61.31 ± 16.93a	6.06	.002
	Middle (4.6~< 10.4 million)	618 (30.5)	247 (25.6)	61.21 ± 17.14a			371 (34.9)	60.84 ± 15.37a		
	High (≥ 10.4 million)	849 (41.8)	586 (60.7)	67.42 ± 13.75b		(a < b^†^)	263 (24.7)	64.98 ± 14.67b		(a < b^†^)
Smoking status	Yes	171 (8.4)	160 (16.6)	62.56 ± 14.29	2.35	.019	11 (1.0)	57.27 ± 14.89	1.00	.318
	No	1,857 (91.6)	805 (83.4)	65.63 ± 15.23			1,052 (99.0)	62.10 ± 15.94		
Alcohol consumption	Yes	654 (32.2)	495 (51.3)	66.65 ± 14.23	−3.23	.001	159 (15.0)	61.70 ± 15.27	0.30	.762
	No	1,374 (67.8)	470 (48.7)	63.51 ± 15.84			904 (85.0)	62.11 ± 16.05		
Number of chronic diseases	≥ 2	887 (43.7)	351 (36.4)	63.48 ± 15.41a	3.95	.020	536 (50.4)	60.47 ± 15.93a	6.64	.001
	1	653 (32.2)	316 (32.7)	66.74 ± 14.86b			337 (31.7)	62.85 ± 16.43		
	0	488 (24.1)	298 (30.9)	65.34 ± 14.86		(a < b^†^)	190 (17.9)	65.11 ± 14.50b		(a < b^†^)

Values are presented as the mean ± standard deviation or n (%). KRW = Korean won.

† Scheffé's test.

**Table 2.** Sensitivity Analysis of the Association between Frailty and Quality of Life across Different Cut-off Thresholds (N = 2,028)

Variables	Cut−off	B	95% CI	*p*
Overall frailty	≥ 4	−3.71	(−5.02, −2.40)	< .001
	≥ 5	−4.90	(−6.73, −3.08)	< .001
	≥ 6	−7.25	(−9.53, −4.98)	< .001
Physical frailty	≥ 2	−2.32	(−3.18, −1.47)	< .001
	≥ 3	−3.50	(−4.84, −2.16)	< .001
	≥ 4	−3.97	(−6.01, −1.93)	< .001
Psychological frailty	≥ 1	−4.49	(−5.83, −3.14)	< .001
	≥ 2	−4.70	(−6.92, −2.49)	< .001
	≥ 3	−4.34	(−8.04, −0.65)	.021
Social frailty	≥ 1	−1.78	(−2.56, −1.01)	< .001
	≥ 2	−1.74	(−3.11, −0.37)	.013
	≥ 3	−2.64	(−4.58, −0.71)	.005

Covariates included in the model: age, education level, presence of spouse, residential area, religion, total income, smoking status, alcohol consumption, and number of chronic diseases. CI = Confidence interval.

**Table 3.** Associations between Physical, Psychological, and Social Frailty and Quality of Life by Sex (N = 2,028)

Variables		Men				Women			
		B	SE	95% CI	*p*	B	SE	95% CI	*p*
Overall frailty	No	ref.				ref.			
	Yes	−7.98	1.28	(−10.48, −5.48)	< .001	−9.42	0.89	(−11.16, −7.68)	< .001
Physical frailty	No	ref.				ref.			
	Yes	−3.02	1.01	(−5.00, −1.03)	.003	−6.88	0.72	(−8.30, −5.46)	< .001
Psychological frailty	No	ref.				ref.			
	Yes	−8.51	1.71	(−11.87, −5.16)	< .001	−5.88	1.32	(−8.48, −3.28)	< .001
Social frailty	No	ref.				ref.			
	Yes	−2.53	1.21	(−4.90, −0.17)	.035	−2.61	0.80	(−4.18, −1.04)	.001

Covariates included in the model: age, education level, presence of spouse, residential area, religion, total income, smoking status, alcohol consumption, and number of chronic diseases. SE = Standard error; CI = Confidence interval; ref. = Reference category.

## Data Availability

The data that support the findings of this study are available from the corresponding author upon reasonable request.
